# CAGO: A Software Tool for Dynamic Visual Comparison and Correlation Measurement of Genome Organization

**DOI:** 10.1371/journal.pone.0027080

**Published:** 2011-11-17

**Authors:** Yi-Feng Chang, Chuan-Hsiung Chang

**Affiliations:** 1 Center for Systems and Synthetic Biology, National Yang-Ming University, Taipei, Taiwan; 2 Institute of Biomedical Informatics, National Yang-Ming University, Taipei, Taiwan; University of Minnesota, United States of America

## Abstract

CAGO (Comparative Analysis of Genome Organization) is developed to address two critical shortcomings of conventional genome atlas plotters: lack of dynamic exploratory functions and absence of signal analysis for genomic properties. With dynamic exploratory functions, users can directly manipulate chromosome tracks of a genome atlas and intuitively identify distinct genomic signals by visual comparison. Signal analysis of genomic properties can further detect inconspicuous patterns from noisy genomic properties and calculate correlations between genomic properties across various genomes. To implement dynamic exploratory functions, CAGO presents each genome atlas in Scalable Vector Graphics (SVG) format and allows users to interact with it using a SVG viewer through JavaScript. Signal analysis functions are implemented using R statistical software and a discrete wavelet transformation package waveslim. CAGO is not only a plotter for generating complex genome atlases, but also a platform for exploring genome atlases with dynamic exploratory functions for visual comparison and with signal analysis for comparing genomic properties across multiple organisms. The web-based application of CAGO, its source code, user guides, video demos, and live examples are publicly available and can be accessed at http://cbs.ym.edu.tw/cago.

## Introduction

Genome atlas plotters (also known as genome diagram plotters or chromosome atlas plotters) are usually designed to plot genomic features (positional annotations) and genomic properties (numerical values) of a genome as chromosome tracks in a static picture (Table S1). However, a static genome atlas does not provide the functions of dynamic exploratory and signal analysis for genomic properties.

We developed CAGO (Comparative Analysis of Genome Organization) to address these shortcomings by integrating dynamic exploratory functions into a genome atlas tool and implementing signal analysis functions to analyze genomic properties. The dynamic exploratory functions are not like the navigating and zooming functions of conventional genome browsers, but are designed to interactively manipulate each individual track of a genome atlas by modifying its image attributes. The image attributes include track position, angle of a circular track, color opacity, track width, and image mirroring. For example, users can change the color opacity of tracks and reposition a track onto other tracks with the interactive functions, and then compare similarities or differences between different genomic features or genomic properties by visual comparison. With signal analysis functions users can reveal the global identity of a noisy genomic property by denoising functions, such as the discrete wavelet transformation (DWT) [1]. In addition, users can identify inconspicuous periodic patterns from a genomic property by autocorrelation [2] and calculate correlations between different genomic properties across multiple organisms by cross-correlation analysis [3].

A genomic property is also a kind of waveform signal. Thus, wavelet transformation is a useful method to analyze genomic properties [4]–[13]. To extract the essence of a noisy genomic property, discrete wavelet transformation is implemented to decompose a genomic property into different scales of signal frequencies. A smaller scale of signal frequencies represents the noise parts of a waveform signal, while a larger one represents a global identity of the waveform signal. The denoised version of a noisy genomic property can be reconstructed from the decomposed signals [14].

Autocorrelation can detect rhythmic patterns from a genomic property, for examples, identifying spatial periodic patterns of gene expression activity in bacterial chromosome [15], [16] and detecting sequence periodicity of chromosomes [17]–[20]. The concept of detecting periodic patterns is to compare a genomic property with its phase-shifted versions at all positions, therefore, the output of autocorrelation analysis is a series of correlation coefficients. If a genomic property has a rhythmic pattern occurred at a specific period, a high correlation peak can be identified at the position of that specific period from the output of autocorrelation analysis. However, if no correlation peak is found in the autocorrelation output of a genomic property, the property is considered as a random signal. Similar to the concept of autocorrelation, cross-correlation is used to calculate the degree of similarity between a phase-fixed genomic property and another phase-shifted genomic property at all positions. The output of cross-correlation is also a series of correlation coefficients. If two genomic properties are similar to each other, a high correlation peak can be found at a specific position in the output of cross-correlation analysis. This means that by sliding the phase-shifted version of genomic property to the specific position, the overlapped regions of the two genomic properties can become similar.

The web-based application of CAGO is available at http://cbs.ym.edu.tw/cago, including source code, detailed user guides, video demos, and examples used in this publication. The source code of CAGO has also been published as an open source project at Google Code (http://code.google.com/p/cago/) under a GNU GPL license.

## Results and Discussion

In our web-based CAGO, 222 (149 for eukaryotic sequences) genomic features and genomic properties (See Table S2) are applied to the sequences downloaded from the NCBI ftp site [21]. These sequences include 1,562 prokaryotic chromosomes, 2,398 prokaryotic plasmids, 227 eukaryotic chromosomes, 3,140 virus genomes, 615 bacteriophage genomes, 2,518 mitochondrial sequences, and 202 plastid sequences (downloaded on August 21, 2011).

### User interfaces of CAGO

#### Customized Track Uploader

**Figure 1:**
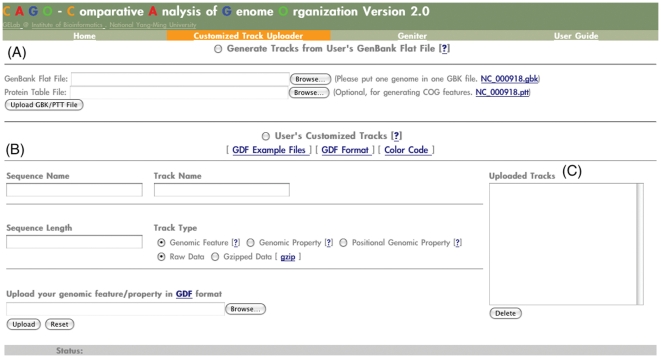
Screenshot of Customized Track Uploader. (A) For uploading a GenBank flat file and a protein table file of a sequence. The CAGO pipeline can automatically generate all genomic feature tracks and genomic property tracks for the sequence. (B) For uploading users' customized tracks. A customized track must be written in Geniter data format (GDF; see online user guide for further details.) Together with a sequence name, a sequence length, a track name, and a track type, users can upload the customized track to CAGO. (C) For listing and deleting uploaded and generated chromosome tracks. Users can only see or delete chromosome tracks uploaded by themselves.

Besides providing pre-computed genomic features and genomic properties of a genome, we want our users to be able to create and view additional features that can aid in their genomic studies. Therefore, we developed the Customized Track Uploader for users to upload their genome sequences, customized genomic features, and customized genomic properties (Figure 1). An automatic pipeline is used to produce all pre-defined genomic features and genomic properties for the uploaded sequences. The genomic features and genomic properties generated by the pipeline and the customized data can then be used in a genome atlas configuration interface. To protect data privacy, a browser session is kept in cookies and stored in the client-side computer, users can only access the data uploaded by themselves.

#### Genome Atlas Configurator (Geniter)

**Figure 2:**
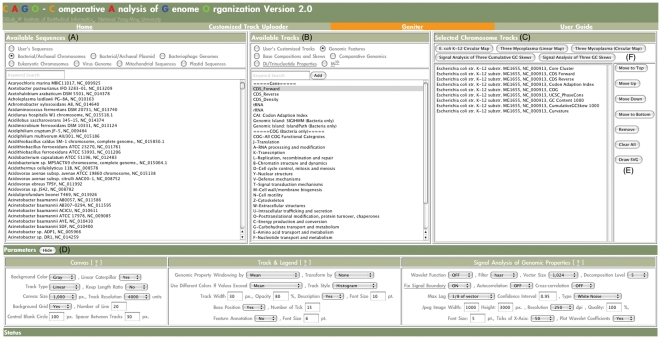
Screenshot of Genome Atlas Configurator. (A) Available Sequences. Sequences uploaded by users and sequences processed by the CAGO pipeline are listed in this panel. (B) Available Tracks. User's customized tracks, genomic feature tracks, and genomic property tracks provided by CAGO are listed in this panel. (C) Selected Chromosome Tracks. To plot a genome atlas, at least one chromosome track must be selected. By choosing a sequence and a track and then click the “Add” button, a chromosome track can be added to the list of “Selected Chromosome Tracks.” (D) The canvas parameters of a genome atlas, the parameters of chromosome tracks and legends, and the parameters for signal analysis of genomic properties. See the online user guide for further details of the parameters (http://cbs.ym.edu.tw:8080/CAGO/userguide.html.). (E) Chromosome track arrangement buttons. Users can use the buttons to modify the order of selected chromosome tracks of a genome atlas and to remove one or multiple chromosome tracks from a genome atlas. (F) Examples used in this publication. The buttons of “Three *Mycoplasma* (Linear Map)” and “Three *Mycoplasma* (Circular Map)” can be used to reproduce Figures 2A and 2B. The button of “*E. coli* K-12 Circular Map” can be used to reproduce Figure 3. The buttons of “Signal Analysis of Three Cumulative GC Skews” and “Signal Analysis of Three GC Skews” can be used to reproduce Figure 5 and Figures S1 and S2. By clicking the “Draw SVG” button, the genome atlas of the selected chromosome tracks can be generated in the SVG Genome Atlas Viewer.

Geniter is an interface for configuring a genome atlas (Figure 2). With Geniter, users can select genomic features and genomic properties of one or many organisms. To produce a genome atlas, track parameters such as opacity, track style, and track width, can be configured individually for each chromosome track. A set of pre-defined settings is given when using Geniter, and users can adjust the parameters to produce more complex genome atlas. The SVG-based genome atlas will then be generated and presented in our SVG Genome Atlas Viewer.

#### SVG Genome Atlas Viewer

**Figure 3:**
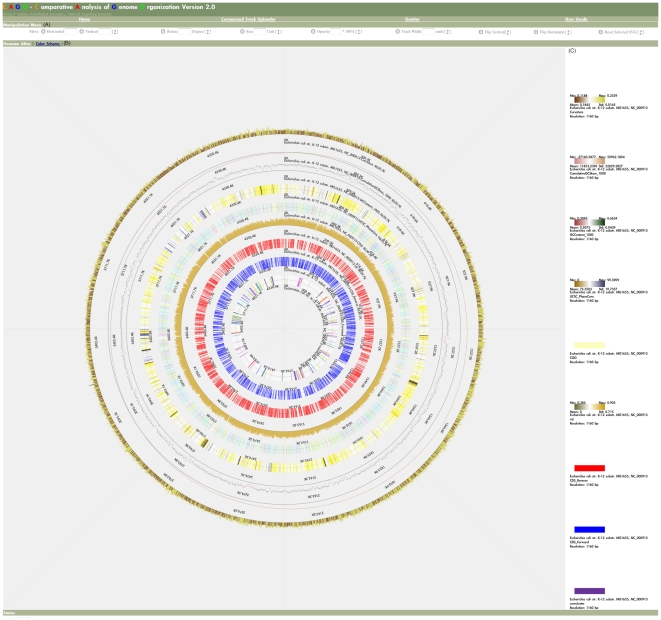
Screenshot of SVG Genome Atlas Viewer. (A) The operation menu for dynamic exploratory functions (B) The genome atlas of *E. coli* with nine chromosome tracks: (1) bacteria-specific core genes of *E. coli*, (2) forward and (3) reverse strands of CDSs, (4) codon adaptation index, (5) COGs of CDSs, (6) sequence conservation data downloaded from UCSC archaeal genome browser, (7) GC percentage (window size = 1 kb), (8) cumulative GC skew (window size = 1 kb) and (9) DNA Curvature. (C) The legends for the nine chromosome tracks.

SVG Genome Atlas Viewer is not only an interface for presenting a genome atlas but also an interface for manipulating chromosome tracks. To demonstrate the basic presentation function of SVG Genome Atlas Viewer, the genomic features and genomic properties of *Escherichia coli* (*E. coli*) str. K-12 substr. MG1655 were plotted as circular tracks (Figure 3). The tracks arranged from the inner circle to the outer circle are as follows: (1) bacteria-specific core genes of *E. coli* (customized data), (2) forward and (3) reverse strands of coding sequences (CDSs), (4) codon adaptation index [22], (5) functional categories of clusters of orthologous groups (COGs) of CDSs [23], (6) sequence conservation data (customized data) downloaded from UCSC archaeal genome browser [24], (7) GC percentage (window size = 1 kb), (8) cumulative GC skew (window size = 1 kb), and (9) DNA Curvature [25]. In this example, all the default settings were used and only the track resolutions were modified to 4,000 units.

The operation menu of dynamic exploratory functions is placed at the top of the viewer (Figure 3A). The SVG canvas of nine chromosome tracks is placed in the Genome Atlas panel (Figure 3B). Legends for all chromosome tracks are listed at the right-hand side of the Genome Atlas (Figure 3C). The min, max, mean, and standard deviation of a genomic property are placed around the four corners of its legend. The window size used to condense a genomic feature or a genomic property into a chromosome track is placed below its legend. For instance, in the case of DNA Curvature, a sliding window with size of 1,160 bp was used to condense the original Curvature property into 4,000 units (4639 kb/1.16 kb).

In CAGO, tracks of genomic features are presented in solid colors (track 1, track 2, track 3, and track 5); and tracks of genomic properties can be presented in three styles: histograms (track 4 and track 9), data dots (track 8 and track 7) and gradient colors (track 6). Blue and red colors are used to indicate the forward and reverse CDSs (track 2 and track 3), respectively. The colors used to represent different COG categories (track 5) are adopted from NCBI COG website [26]. If positional annotations of a customized genomic feature have no color code assigned before upload, the solid colors of the positional annotations are assigned randomly from a pre-defined color palette (track 1) by SVG Genome Atlas Viewer. For a track that is presented in histogram or data dot, a two-color scheme is used to indicate whether values of a genomic property exceeded a certain value such as mean. For a track using gradient colors, values of a genomic property are converted to corresponding colors. And the darkest colors on the both ends of a two-color scheme are used to indicate the minimum (leftmost) and the maximum (rightmost) values of a genomic property.

### Dynamic exploratory functions for visual comparison

**Figure 4:**
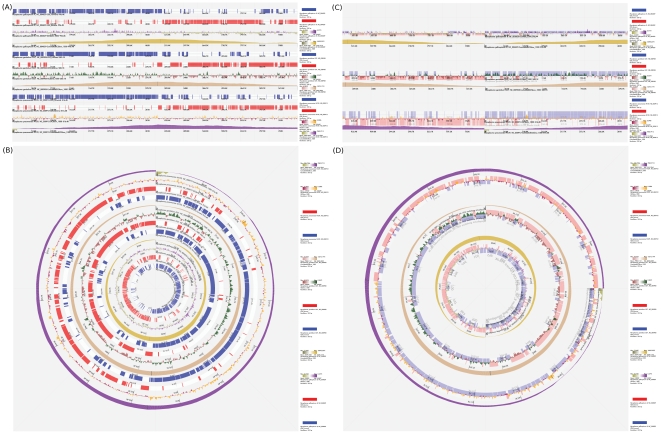
Demonstrations of dynamic exploratory functions for visual comparison. Figures 4A and 4B are the original genome atlases of the linear and circular chromosome tracks for the forward and reverse strands of CDSs, GC skews (window size = 1 kb) and cumulative GC skews (window size = 1 kb) of the chromosomes of *M. gallisepticum*, *M. genitalium*, and *M. pneumonia*. The results after applying the track manipulation procedures on the chromosome tracks in Figures 4A and 4B are shown in Figures 4C and 4D, respectively.

To demonstrate the dynamic exploratory functions for manipulating linear and circular tacks in SVG Genome Atlas Viewer, four kinds of chromosome tracks were plotted in linear (Figure 4A) and circular tracks (Figure 4B) for three *Mycoplasma* species, including *Mycoplasma gallisepticum* R (*M. gallisepticum*), *Mycoplasma genitalium* G-37 (*M. genitalium*), and *Mycoplasma pneumoniae* M129 (*M. pneumonia*). The four chromosome tracks demonstrated here are, from top to bottom and from inner circle to outer circle, forward and reverse strands of CDSs, GC skew (window size = 1 kb), and cumulative GC skew (window size = 1 kb).

The configurations of the linear genome atlas (Figure 4A) were as follows: (1) linear track type; (2) canvas size of 2000 units; (3) track resolution of 3000 units; (4) 45 units of spacer size between tracks. The parameters of the circular genome atlas (Figure 4B) were as follows: (1) circular track type; (2) canvas size of 1000 units; and (3) track resolution of 3000 units. To demonstrate scale-independent organizations of the three *Mycoplasma* species, ratios of all chromosome lengths were discarded.

The manipulation procedures of Figure 4A were as the followings. First, all chromosome tracks were repositioned by aligning the beginning of the tracks to the center of genome atlas. To present a circular chromosome in linear track, a display function called caterpillar can make a linear chromosome track to have a circular rotation effect. To elaborate the caterpillar function, when a linear track is repositioned horizontally to the leftmost side of the canvas and beyond the boundary of canvas, the leftmost side of the track would disappear. At the same time, the disappeared region is shown up from the rightmost side of the canvas. Due to the caterpillar function all linear tracks were remained intact after the manipulation (Figure 4C). Second, the color opacity of all forward and reverse strands of CDSs was changed to 30%. Third, the track widths of forward and reverse strands of CDSs for *M. gallisepticum*, *M. genitalium*, and *M. pneumoniae* were changed to 15, 30, and 50 units, respectively. Finally, the forward and reverse strands of CDSs of each organism were repositioned onto its GC skew track. The final result of the chromosome track manipulation is shown in Figure 4C.

The manipulation procedures of Figure 4B were as the followings. First, the color opacity of all forward and reverse strands of CDSs was changed to 30%. Second, all chromosome tracks of *M. pneumoniae* were rotated 90 degrees clockwise. Third, all chromosome tracks of *M. gallisepticum* were flipped vertically. Fourth, all chromosome tracks of *M. genitalium* were flipped horizontally. Finally, the diameters of the circular tracks of forward and reverse strands of CDSs of each organism were increased to overlap onto its GC skew track. The final result of the chromosome track manipulation is shown in Figure 4D.

The plots of GC skew [(G−C)/(G+C)] and the cumulative GC skew (the sum of GC skew) of a prokaryotic chromosome have been used to identify the origins of replication (oriC) [27]–[35]. Over 70% of the completely sequenced prokaryotic chromosomes have GC skew polarities, that is, the number of nucleotide G is always more abundant than that of nucleotide C in the replication leading strand in a prokaryotic chromosome. Prokaryotic chromosomes that do not have GC skew polarities are those of thermophiles, cyanobacteria and *Deinococcus radiodurans* [34]. If a chromosome has GC skew polarities, two polarities can be observed in its GC skew plot. The replication origin and terminus of a chromosome can be identified at the switch points of the two polarities. A detailed review of identifying prokaryotic replication origins can be found in Reference [36]. Furthermore, if a chromosome has GC skew polarities, the plot of cumulative GC skew can form a Λ-shaped, V-shaped or shifted Λ/V-shaped curve. The formation of a Λ-shaped, V-shaped or shifted Λ/V-shaped cumulative GC skew plot depends on whether the starting base of a chromosome is the replication origin, the replication terminus, or an arbitrary position. For a chromosome that has a plot of Λ- or V-shaped cumulative GC skew, the positions of minimum and maximum values of a cumulative GC skew usually coincide with the sites of replication origin and terminus of the chromosome, respectively. However, exceptions are found in the chromosomes of *Streptomyces coelicolor* A3 (2) and *Streptomyces griseus* subsp. griseus NBRC 13350. The replication origins of the two *Streptomyces* are at the center of chromosomes where the maximum cumulative GC skews are. To improve the accuracy of predicting the origins of replication in prokaryotic chromosomes, many genomic signals, such as mononucleotide skews (such as AT skew), dinucleotide skews (such as keto skew), oligonucleotide skews, and gene strand skews can be combined together [34], [36]. In addition, replication-associated genomic features such as the locations of *dnaA* gene and rRNA operons [32], [37], the positions of Chi (crossover hotspot instigator) motifs, *parS* motifs and motifs regarding segregation of replication origin should be good criteria for identifying the replication origins of prokaryotic chromosomes [38].

In Figure 4, through the dynamic exploratory functions, users can manipulate chromosome tracks and compare the global arrangements of different tracks across multiple organisms. Although the chromosome lengths of the three *Mycoplasma* genomes are different, they all share scale-independent genome organizations. Their genes are majorly encoded in replication leading strands, and most of coding sequences are enriched in high GC skews. In addition, all of their chromosomes have GC skew polarities and all have Λ-shaped cumulative GC skews. This indicates that despite the low GC contents of the chromosomes of the three *Mycoplasma* species, they use more nucleotide G than nucleotide C in the replication leading strands. In the three *Mycoplasma* genomes, the replication origins are at the beginnings of DNA sequences, which are confirmed by the locations of annotated *dnaA* genes of the three *Mycoplasma* species and by the experimental results from other studies [33]–[35].

### Signal analysis of cumulative GC skews

**Figure 5:**
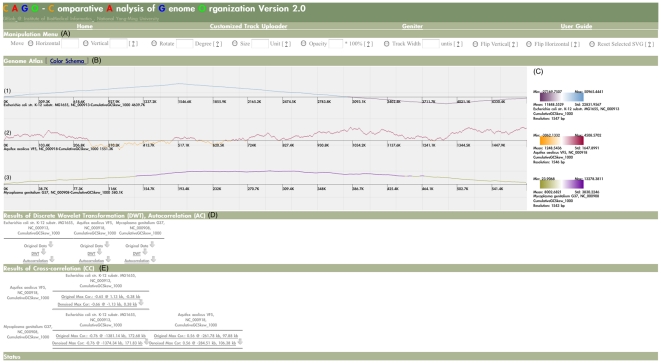
A screenshot of signal analysis results for the cumulative GC skews (window size = 1 kb) of the chromosomes of *E. coli*, *A. aeolicus* and *M. genitalium*. (A) The operation menu for dynamic exploratory functions. (B) The genome atlas of the cumulative GC skews (window size = 1 kb) of the chromosomes of (1) *E. coli*, (2) *A. aeolicus*, and (3) *M. genitalium*. (C) The legends for the three chromosome tracks. (D) Results of discrete wavelet transformation and autocorrelation for the three cumulative GC skews. (E) Results of pair-wise cross-correlation for the three cumulative GC skews.

To demonstrate signal analysis of genomic property, the cumulative GC skews (window size = 1 kb) calculated from the chromosomes of *E. coli* str. K-12 substr. MG1655, *Aquifex aeolicus* (*A. aeolicus*) VF5, and *Mycoplasma genitalium* (*M. genitalium*) G37 were used. The reason of choosing the three cumulative GC skews is that the three curves have different shapes of skews. The screenshot of the example is illustrated in Figure 5. The plot of the cumulative GC skew of *E. coli* is a shifted Λ-shaped curve (Figure 5B-1), and the plot of *M. genitalium* is a Λ-shaped curve (Figure 5B-3). The positions of the minimum cumulative GC skews of chromosomes of *E. coli* str. K-12 substr. MG1655 and *M. genitalium* G37 are located at 3923.4 kb and 1 bp, respectively. The two positions also coincide with the replication origins of the two chromosomes. However, the plot of the cumulative GC skew of *A. aeolicus* chromosome (Figure 5B-2) has no clear V-shaped pattern. Although the predicted oriC of the chromosome of *A. aeolicus* is located at 209 kb based on the DORIC database [35], the minimum cumulative GC skew is located at 343.3 kb. Without a clear V-shaped cumulative GC skew indicates that the GC skew of *A. aeolicus*, a hyperthermophile, has no GC skew polarities. This might be resulted from the fact that the chromosomes of hyperthermophiles use different strategies to protect DNA from extreme thermal environments compare to the strategies used by mesophiles that have GC skew polarities [39]. Another speculation of why thermophiles have no GC skew polarities is that the nucleotide skews of thermophiles might be influenced by DNA polymerases they used [34].

The parameters used for this example were as follows: (1) linear track type; (2) ratios of lengths were discarded; (3) canvas size of 1200 units; (4) track style of 1 pixel dot; (5) track resolution of 3000 units; (6) discrete wavelet transformation, autocorrelation and cross-correlation were turned on; (7) Haar wavelet filter; (8) confidence interval at 99.9%; (9) vector size of 2,048; (10) maximum decomposition level was four; (11) maximum lag was the same as vector size; (12) JPEG image size of 1000×3000 pixel; (13) JPEG resolution of 300 dots per inch; (14) JPEG font size of 15 point.

As discrete wavelet transformation, autocorrelation and cross-correlation were applied to analyze genomic properties, two additional panels were appended to the bottom of SVG Genome Atlas Viewer (Figures 5D and 5E). The resulting images of discrete wavelet transformation and autocorrelation for each genomic property are placed in the first panel. Users can access these images through hyperlinks named after “DWT” and “Autocorrelation” (Figure 5D). The resulting images of cross-correlation between any two mutually distinct entities of selected genomic properties are put in the second panel as hyperlinks named after their maximum correlations (Figure 5E). CAGO only calculates half of the pairwise results of cross-correlation while comparing selected genomic properties because the results of pairwise cross-correlation are symmetric. In addition, autocorrelation and cross-correlation can be used to test whether the wavelet coefficients and the denoised versions of the selected genomic properties have periodic patterns and whether they are similar to each other. The selected genomic properties and the results of discrete wavelet transformation, autocorrelation, and cross-correlation can be downloaded for further study through the button next to the hyperlinks.

#### Discrete Wavelet Transformation

**Figure 6:**
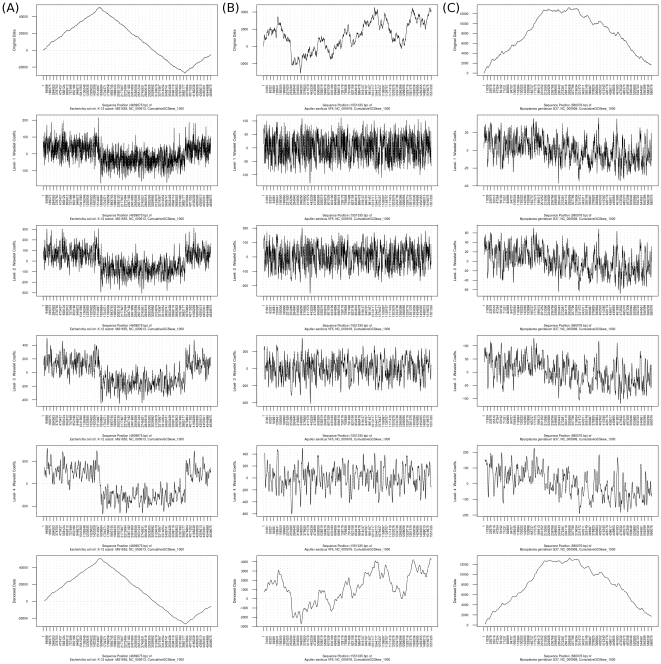
Results of discrete wavelet transformation for the cumulative GC skews of the chromosomes of *E. coli*, *A. aeolicus* and *M. genitalium*. Figure 6 shows the plots of original cumulative GC skews, the plots of level-1 to level-4 wavelet coefficients, and the plots of denoised versions of the cumulative GC skews of the chromosomes of (A) *E. coli*, (B) *A. aeolicus* and (C) *M. genitalium*, respectively. The X-axis represents the sequence positions of each organism. The Y-axis represents the values of a cumulative GC skew or the values of wavelet coefficients.

The DWT results of the cumulative GC skews of *E. coli*, *A. aeolicus* and *M. genitalium* are shown in Figure 6. Six images were generated for each genomic property since the maximum decomposition level used in this example was four. The first image is the original plot of a cumulative GC skew, and the second to the fifth images are plots of wavelet coefficients of a cumulative GC skew from level one to four. These wavelet coefficients also indicate the noise parts of a cumulative GC skew captured by discrete wavelet transformation at different levels. By reconstructing the highest level of wavelet coefficients of a cumulative GC skew, a denoised version of the cumulative GC skew is plotted in the last image.

Wavelet coefficients at each level can be used to describe the characteristics of a genomic property at different scales of frequencies. Level-one wavelet coefficients represent the highest frequencies (noise) of a genomic property; and higher-level wavelet coefficients (global identity) indicate lower frequencies of a genomic property. For example, in the wavelet coefficients of cumulative GC skew of *E. coli* (Figure 6A), two polarities (above zero and below zero) became clear when the decomposition level increases from level one to level four. The two switch points of the polarity pattern also coincided with an obvious oscillation pattern of the original *E. coli* cumulative GC skews, i.e. two vertices (the highest and the lowest peaks) in the shifted Λ-shaped curves.

A cumulative GC skew is the sum of GC skew of a window sliding along a sequence [29]. The plot of wavelet coefficients of a cumulative GC skew captured by DWT with Haar wavelet filter at a specific decomposition level is similar to a plot of denoised GC skew reconstructed from wavelet coefficients of the original GC skew at the same decomposition level. This phenomenon is caused by the characteristics of Haar wavelet filter. When using DWT with Haar wavelet filter to decompose a cumulative signal (e.g. cumulative GC skew) into wavelet coefficients, the plot of the wavelet coefficients is similar, but not identical, to a denoised plot of its reverse cumulative signal (e.g. GC skew) at the same decomposition level. However other wavelet filters, except FK4 (Fejer-Korovkin) wavelet filter, do not have similar reverse cumulative function found in the Haar wavelet filter. Detailed descriptions of different kinds of wavelet filters can be found in Reference [40]. Thus, the plot of level-one wavelet coefficients of the cumulative GC skew of *E. coli* is similar to a plot of denoised GC skew reconstructed from the level-one wavelet coefficients of the GC skew of *E. coli* (r = 0.96, n = 2,048, P<2.2e^−16^, 95% CI 0.96 to 0.97). In addition, the plot of level-four wavelet coefficients of cumulative GC skew of *E. coli* is similar to a plot of denoised GC skew reconstructed from the level-four wavelet coefficients of the GC skew (r = 0.97, n = 2,048, P<2.2e^−16^, 95% CI 0.97 to 0.98; see Figure S1A). The same observations also apply to the plots of wavelet coefficients of the cumulative GC skew of *A. aeolicus* (Figure 6B and Figure S1B) and to the plots of wavelet coefficients of the cumulative GC skew of *M. genitalium* (Figure 6C and Figure S1C). With DWT analysis, the essence of the three cumulative GC skews can be extracted as wavelet coefficients at different scales; and denoised versions of these genomic properties can be reconstructed. There is no significant difference between the original versions and the denoised versions of the plot of cumulative GC skews of *E. coli*. The original and the denoised versions of the plot of cumulative GC skew of *M. genitalium* are similar to each other. This is because their cumulative GC skew curves are not noisy. However, differences can be found between the plots of the original and the denoised versions of the cumulative GC skew of *A. aeolicus* after the noise parts are removed from the cumulative GC skew by discrete wavelet transformation.

#### Autocorrelation

**Figure 7:**
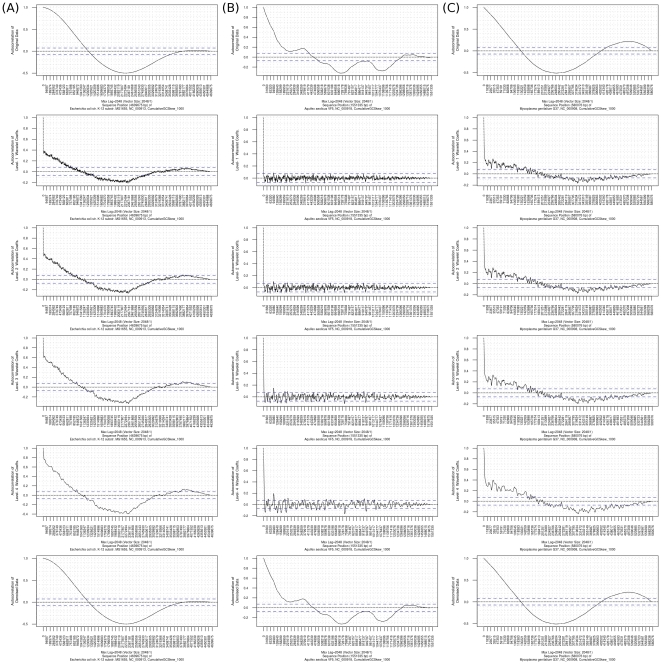
Results of autocorrelation analysis for the cumulative GC skews of the chromosomes of *E. coli*, *M. genitalium* and *A. aeolicus*. Figure 7 shows the results of autocorrelation analysis for the original cumulative GC skews, the level-1 to level-4 wavelet coefficients and the denoised versions for the cumulative GC skews of the chromosomes of (A) *E. coli*, (B) *A. aeolicus* and (C) *M. genitalium*. The X-axis represents the shifting lags (sequence positions) of each organism. The Y-axis represents the degrees of autocorrelation coefficients at different lags. The two horizontal blue lines are the 99.9% of confidence interval (0.073).

The results of autocorrelation of three cumulative GC skews are shown in Figure 7. From the results of the original cumulative GC skews of *E. coli* and *M. genitalium*, the highest correlation peaks are at 2,319.8 kb (−0.50) and 255.2 kb (−0.51), respectively. In other words, periodic patterns in mirroring phase can be found in the cumulative GC skew of *E. coli* for every 2319.8 kb and in *M. genitalium* for 255.2 kb (The first images of Figures 7A and 7C). In addition, in the denoised cumulative GC skews of *E. coli* and *M. genitalium*, the highest correlation peaks (−0.5) are at the same positions of the output (The last images of Figures 7A and 7C). The positions of the highest correlation peaks in the autocorrelation results are about half of the whole length of chromosomes of *E. coli* and *M. genitalium*, which also coincide with the distances from the minimum to the maximum cumulative GC skews. This indicates that the autocorrelation function can detect the 1 Hz periodic patterns of the plots of the shifted Λ-shaped and Λ-shaped cumulative GC skews of *E. coli* and *M. genitalium*. In *A. aeolicus*, although the highest correlation peaks are at the lags of 713.6 kb in the original and the denoised cumulative GC skews, the maximum correlations are not strong (−0.32) (the first and the last images of Figure 7B). On the other hand, by applying autocorrelation analysis to wavelet coefficients, we can estimate if the noise parts of a genomic property have periodic patterns. For example, in the level-four wavelet coefficients of cumulative GC skew of *A. aeolicus*, the highest correlation peak is at the lag of 93 kb (Figure 7B). The plot of level-four wavelet coefficients of the cumulative GC skew of *A. aeolicus* is similar to a plot of denoised GC skew reconstructed from the level-four wavelet coefficients of the GC skew of *A. aeolicus* (Figure S2B). Therefore a correlation peak at the position of 93 kb can be identified in the denoised version of the GC skew of *A. aeolicus* but the correlation is not strong (−0.21). In the autocorrelation output of the original GC skew of *A. aeolicus*, no significant autocorrelation peak was identified, which means that the original GC skew might be a random signal (Figure S2B). However, the finding of the 93 kb autocorrelation peak in the level-four wavelet coefficients of cumulative GC skew of *A. aeolicus* (Figure 7B) indicates that the reconstructed GC skew has a stable, although weak (low correlation coefficient) structure. Although *A. aeolicus* has no GC skew polarity, the stable periodic pattern might suggest that, in addition to the strategies used to stabilize DNA, hyperthermophiles may use different nucleotide compositions in their chromosomes to maintain the chromosome stability in extreme thermal environments [39].

#### Cross-correlation

**Figure 8:**
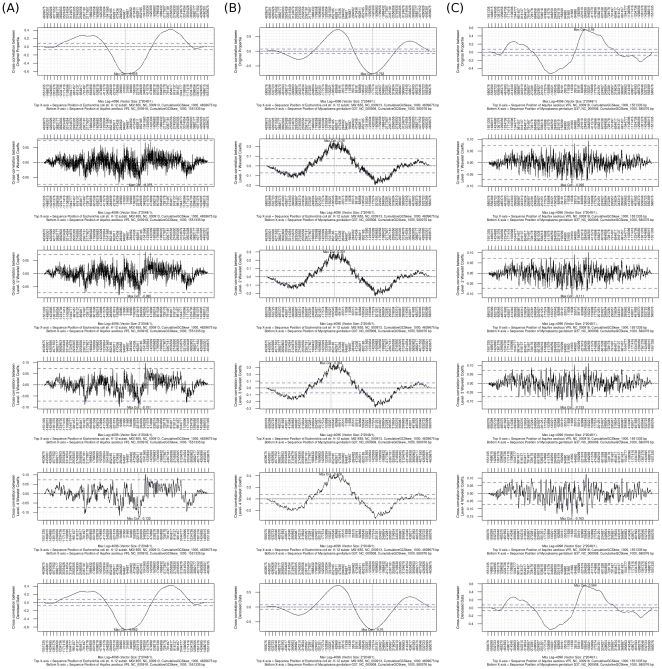
Results of cross-correlation analysis for the three cumulative GC skews of the chromosomes of *E. coli*, *M. genitalium* and *A. aeolicus*. Figure 8 shows the results of pairwise cross-correlation for the cumulative GC skews of the chromosomes of (A) *E. coli* and *A. aeolicus*, (B) *E. coli* and *M. genitalium*, and (C) *A. aeolicus* and *M. genitalium*. In order to estimate all possible conditions of cross-correlation between two genomic properties, the lags used in this example are ranged from maximum negative lag to maximum positive lag. Lags may indicate different positions of two genomic properties because cross-correlation compares two independent genomic properties that may have different sequence lengths. Therefore, two X-axes are used to label the shifting *lags* (sequence positions) of two genomic properties. The top X-axis represents the sequence positions of a phase-fixed genomic property; the bottom X-axis represents the sequence positions of a phase-shifted version of genomic property. The Y-axis represents the degrees of cross-correlation coefficients at different lags. A vertical solid gray line is used to point out the position of the maximum correlation of two genomic properties. The two horizontal blue lines are the 99.9% of confidence interval (0.073).

The outputs of cross-correlation analysis of three pairs of cumulative GC skews are shown in Figure 8. The maximum correlations between *E. coli* and *A. aeolicus* (Figure 8A), *E. coli* and *M. genitalium* (Figure 8B), and *A. aeolicus* and *M. genitalium* (Figure 8C) are −0.65, −0.76, and 0.56, respectively. For the pair of *E. coli* and *A. aeolicus*, the position of maximum correlation is close to phase zero [1.13 kb (*E. coli*) and −0.38 kb (*A. aeolicus*)], which means while comparing the two genomic properties without changing the shifting phase the maximum correlation of −0.65 can be obtained. To obtain a maximum correlation between the pair of *E. coli* and *M. genitalium*, the phase of the plot of the chromosome of *M. genitalium* has to move rightward 172.68 kb; or the phase of the plot of the chromosome of *E. coli* has to move leftward 1381.14 kb. Thus, in the pair of *A. aeolicus* and *M. genitalium*, the maximum correlation occurred at the shifting phases of −261.78 kb and 97.88 kb. The reason of using cross-correlation to estimate the similarity between two genomic properties is that the beginning positions of two chromosomes might not be defined with the same criterion. Some genomes use the upstream non-coding regions of *dnaA* genes as the first base of chromosome sequences, but others do not use this rule. With cross-correlation, two genomic properties can be compared without concerning whether the definition of the first base of each chromosome is the same or not.

The correlation between *E. coli* and *A. aeolicus* (−0.65) is similar to that between *E. coli* and *M. genitalium* (−0.76). However, if we visually compare the similarity between the plots of the cumulative GC skews of *E. coli* and *A. aeolicus* with the similarity between the plots of *E. coli* and *M. genitalium*, the similarity between the plot of *E. coli* and the plot of *A. aeolicus* is worse than the similarity between the plot of *E. coli* and the plot of *M. genitalium*. By averaging the maximum correlations of the wavelet coefficients at all levels and the maximum correlation between the denoised versions of two genomic properties, we can conclude that the similarity between the plot of *E. coli* and the plot of *M. genitalium* (0.42) is greater than that between the plot of *E. coli* and the plot of *A. aeolicus* (0.21). To assist the visual comparison, users are advised to combine the results of cross-correlation analyzed from wavelet coefficients of two genomic properties at all levels with the results of cross-correlation calculated from the denoised versions of the two genomic properties.

### Influence of the parameters used in signal analysis

Three parameters can affect the output of signal analysis. They are vector size, wavelet filter, and maximum decomposition level. The vector size of a genomic property directly affects the signal resolution and the computational performance. When condensing a genomic property into a vector to be analyzed by signal analysis, the larger the size of the vector is the better the signal details can be retained. The cost of using a large vector size is time and memory consumption. However, if the vector size of a signal is too small, the condensed vector may lose too many signal details. The balance between computational performance and signal fidelity depends on users' research design. If users are searching for genome-scale patterns from a genomic property, a smaller vector size can be used to ignore minor details of the signal. If users are looking for local-scale patterns, the size of condensed vector should be large enough to represent the details of the original signal. In DWT analysis, the default Haar wavelet filter is one of the simplest wavelet filter, which also has the advantages of less computational and memory usage comparing to other wavelet filters like Daubechies wavelet and Mexican Hat wavelet. In addition, the wavelet coefficients produced by DWT with Haar or FK4 wavelet filter have a property of reversing accumulation procedures of a cumulative signal, which can help users to estimate whether a signal is cumulated from other signals. However, the resolution of the denoised signal processed by Haar wavelet filter is not as good as that of a denoised signal processed by other filters. The Haar wavelet filter should be applied in most cases. If users require denoised genomic properties with higher resolutions, other wavelet filters should be used. An introductory text regarding the differences among wavelet filters and how to choose a wavelet filter for a signal to be analyzed can be found in Reference [40]. On the other hand, the value of maximum decomposition level used in DWT directly affects the numbers of wavelet coefficients generated by DWT and the reconstruction of a denoised signal. If the value is too small, the noise parts of a genomic property cannot be removed. If the value is too large, the details of the denoised signal may be lost. In the discrete wavelet transformation, the value must be less than or equal to log2 (vector size), but there is no gold standard for choosing the value of maximum decomposition level of DWT analysis. Thus, users are encouraged to test different configurations before they stop signal analysis of genomic properties.

The examples discussed in the Results section, and other more complex examples and demonstrations, are available at the CAGO website. Users can load genome atlas configurations of any examples into Geniter with buttons placed above the list of “Selected Chromosome Tracks” (Figure 2F).

## Methods

### System overview of CAGO

CAGO comprises an automatic pipeline, a file-based data repository, a SVG converter, and three web interfaces. The pipeline and the SVG converter are implemented in Java. The three web-based interfaces are implemented in Java Servlet and JavaServe Pages (JSP).

The pipeline uses Biojava [41] to read the GenBank flat file and the protein table of a genome to extract the genomic features like CDSs, COG functional categories of genes, transfer RNA (tRNA) genes, and ribosomal RNA (rRNA) genes. The genomic properties such as base compositions, skews, DNA conformation and thermodynamic properties [42] are calculated from the sequence extracted from the GenBank flat file. This pipeline also uses other bioinformatics tools to produce other genomic features and genomic properties, for instance, genomic islands [43], [44] and codon adaptation index [22]. Pre-computed genomic features and genomic properties are stored in a file-based repository to avoid database dependency.

The SVG converter is responsible for extracting genomic features and genomic properties of genomes selected by users from the repository; and then the converter converts the features and the properties into an SVG-based genome atlas and displays the atlas in the SVG Genome Atlas Viewer. If the signal analysis function is turned on, the converter also converts the genomic properties into vectors and then performs the signal analysis on the vectors.

### Dynamic exploratory functions for visual comparison

The genome atlas generated by CAGO is written in SVG format [45], and the dynamic manipulation functions are implemented in JavaScript to modify the image attributes of SVG elements. Most of the internet browsers embed a SVG viewer and can display a SVG document just like they can display common image file formats, such as JPG and GIF. According to the Document Object Model (DOM) standard [46], objects of a SVG document are considered as HTML objects. Therefore the viewer can respond to users' actions when a mouse or keyboard event is triggered. With the client-side manipulation capability of SVG, users can directly use the mouse cursor to control chromosome tracks. The dynamic exploratory functions of SVG Genome Atlas Viewer are as follows:

1. Move: to reposition a track horizontally or vertically.
2. Rotate: to rotate a circular track clockwise or counterclockwise.
3. Size: to change the size of a track.
4. Opacity: to change color opacity of a track
5. Track width: to change the width of a track wider or narrower
6. Flip: to flip a track vertically or horizontally

To simplify the presentation of a genome atlas, CAGO does not use ribbons to connect related regions (e.g., conserved sequence regions) between different tracks. In addition, when comparing a genomic feature track with a genomic property track of a genome, users can easily observe and compare the relationship between the positional annotations of the genomic feature and the magnitudes of the genomic property by overlapping the two tracks together instead of connecting them with ribbons.

The caterpillar function is implemented with an SVG tag called <USE>. With the element, a chromosome track can be duplicated twice and then put the two replicas on the leftmost and the rightmost flanks of the original chromosome track. With the caterpillar function, a circular chromosome can be displayed in a linear fashion and retain the circular rotation effect.

Besides, to aid in the discovery and comparison of scale-independent genomic signals, CAGO can also proportionally expand smaller genomes and draw chromosome tracks of different sequence lengths from different organisms with as having same lengths.

### Signal analysis of genomic properties

To implement the signal analysis of genomic properties, the SVG converter uses the statistical software R [47] to perform wavelet transformation, autocorrelation and cross-correlation. Given that CAGO is a web-based application, Rserve package [48] and AJAX technology are used to handle multiple requests from different users simultaneously.

To apply wavelet transformation to a genomic property, the discrete wavelet transformation function called *modwt* of the Waveslim package [49], [50] is used. DWT is much more efficient compare with continuous wavelet transformation, because DWT uses powers of two as the number of scales and the number of positions while calculating wavelet coefficients of a genomic property [1]. The first step of decomposing a genomic property is to condense the values of a genomic property into a vector with size of 2*^m^*. In CAGO, *m* is ranged from 10 to 15. The number of scale *J* is ranged from one to the maximum level of *log_2_*(2*^m^*). According to the calculated *J*, DWT can produce *J* groups of wavelet coefficients that decomposed from an original genomic property. All genomic properties are condensed into vectors with the same size of 2*^m^* because cross-correlation requires vectors to be compared in the same sizes.

Autocorrelation can be used to estimate the correlation of a genomic property with a phase-shifted version of itself to detect whether the genomic property contains rhythmic patterns [51]. Autocorrelation calculates the correlation coefficients of all lags. A lag represents the amount of the phase shifted, and the lag starts from zero to 2*^m^* -1). The output of autocorrelation is a series of correlation coefficients with size of 2*^m^*. Similar to autocorrelation, cross-correlation is used to calculate the similarity between two genomic properties by comparing a genomic property with another phase-shifted genomic property [52]. To calculate the correlation at all positions of two genomic properties, the lags of cross-correlation are ranged from -(2*^m^* -1) to (2*^m^* -1). A high correlation peak in the resulting cross-correlation series of two genomic properties indicates that the two properties are correlated at the corresponding lag. To apply autocorrelation and cross-correlation to analyze genomic properties, two functions called *acf* and *ccf* from the stats package of R [47] are adopted in CAGO.

To test the significance of a result of autocorrelation or cross-correlation, we adopted the following equation to determine the confidence interval (*CI*) [47]:
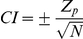
The number *N* is the vector size of a genomic property. *Z_p_* is the number that represents the area under the standard normal distribution curve between - *Z_p_* to *Z_p_* is equal to *p* for a confidence level *p*. For example, *Z_0.95_*, *Z_0.99_*, and *Z_0.999_* are 1.96, 2.58, and 3.29 respectively. A correlation coefficient of autocorrelation or cross-correlation that exceeds a confidence interval (*CI*) is therefore considered significant. The *CI* is plotted as two blue dashed lines in the resulting images of autocorrelation and cross-correlation analysis, adopted from the build-in function of *acf* or *ccf* [47].

### Conclusion

CAGO is developed to improve the functionality of conventional genome atlas viewer by adding the abilities of rapid generation of genome atlas, dynamic exploration of visual comparison and signal analysis of genomic properties. In addition, an automatic pipeline is used to generate genomic features and genomic properties from genome sequences. Several signal analysis examples were used to demonstrate the application of autocorrelation and cross-correlation to identify rhythmic patterns of a genomic property and to estimate the degree of correlation between two genomic properties. Given the ability of chromosome track manipulation and signal analysis of genometric properties, CAGO can assist users to generate and to test their hypotheses regarding genomic research at the global scale.

### GenBank records used in this paper

NC_000908.2: *Mycoplasma genitalium* G-37

NC_000912.1: *Mycoplasma pneumoniae* M129

NC_000913.2: *Escherichia coli* str. K-12 substr. MG1655

NC_000918.1: *Aquifex aeolicus* VF5

NC_003888.3: *Streptomyces coelicolor* A3 (2)

NC_004829.2: *Mycoplasma gallisepticum* R

NC_010572.1: *Streptomyces griseus* subsp. griseus NBRC 13350

## Supporting Information

Figure S1 **Results of discrete wavelet transformation for the GC skews of the chromosomes of** ***E. coli*** **,** ***A. aeolicus*** **and** ***M. genitalium*** **.** Figure S1 shows the plots of original GC skews, the plots of level-1 to level-4 wavelet coefficients, and the plots of the denoised versions of GC skews for the chromosomes of (A) *E. coli*, (B) *A. aeolicus* and (C) *M. genitalium*, respectively. The X-axis represents the sequence positions of each organism. The Y-axis represents the values of a GC skew or the values of wavelet coefficients.
(TIFF)

Figure S2 **Results of autocorrelation analysis for the GC skews of the chromosomes of** ***E. coli*** **,** ***M. genitalium*** **and** ***A. aeolicus*** **.** Figure S2 shows the results of autocorrelation analysis on the original GC skews, the wavelet coefficients and the denoised versions for the GC skews of the chromosomes of (A) *E. coli*, (B) *A. aeolicus* and (C) *M. genitalium*, respectively. The X-axis represents the shifting lags (sequence position) of each organism. The Y-axis represents the degrees of autocorrelation coefficients at different lags. The two horizontal blue lines are the 99.9% of confidence interval (0.073).
(TIFF)

Table S1 **Feature comparison of genome atlas plotters.**
(DOC)

Table S2 **Available genomic features and genomic properties in CAGO.**
(DOC)

## References

[1] Mallat SG (1989). A theory for multiresolution signal decomposition: The wavelet representation.. Pattern Analysis and Machine Intelligence, IEEE Transactions on.

[2] Hamilton JD (1994). Time series analysis.

[3] Orfanidis SJ (1988). Optimum signal processing : an introduction.

[4] Arneodo A, Bacry E, Graves PV, Muzy JF (1995). Characterizing Long-Range Correlations in DNA Sequences from Wavelet Analysis.. Physical Review Letters.

[5] Lio P, Vannucci M (2000). Finding pathogenicity islands and gene transfer events in genome data.. Bioinformatics.

[6] Audit B, Thermes C, Vaillant C, d'Aubenton-Carafa Y, Muzy JF (2001). Long-range correlations in genomic DNA: a signature of the nucleosomal structure.. Physical Review Letters.

[7] Murray KB, Gorse D, Thornton JM (2002). Wavelet transforms for the characterization and detection of repeating motifs.. Journal of Molecular Biology.

[8] Allen TE, Herrgard MJ, Liu M, Qiu Y, Glasner JD (2003). Genome-scale analysis of the uses of the Escherichia coli genome: model-driven analysis of heterogeneous data sets.. Journal of bacteriology.

[9] Audit B, Vaillant C, Arneodo A, d'Aubenton-Carafa Y, Thermes C (2002). Long-range correlations between DNA bending sites: relation to the structure and dynamics of nucleosomes.. Journal of Molecular Biology.

[10] Song J, Ware A, Liu SL (2003). Wavelet to predict bacterial ori and ter: a tendency towards a physical balance.. BMC Genomics.

[11] Nicolay S, Argoul F, Touchon M, d'Aubenton-Carafa Y, Thermes C (2004). Low frequency rhythms in human DNA sequences: a key to the organization of gene location and orientation?. Physical Review Letters.

[12] Allen TE, Price ND, Joyce AR, Palsson B (2006). Long-Range Periodic Patterns in Microbial Genomes Indicate Significant Multi-Scale Chromosomal Organization.. PLoS Computational Biology.

[13] Touchon M, Rocha EPC (2008). From GC skews to wavelets: A gentle guide to the analysis of compositional asymmetries in genomic data.. Biochimie.

[14] Christopher T, Gilbert P (1998). A practical guide to wavelet analysis.. Bulletin of the American Meteorological Society.

[15] Jeong KS, Ahn J, Khodursky AB (2004). Spatial patterns of transcriptional activity in the chromosome of Escherichia coli.. Genome Biology.

[16] Xiao G, Reilly C, Khodursky AB (2009). Improved detection of differentially expressed genes through incorporation of gene locations.. Biometrics.

[17] Trifonov EN, Sussman JL (1980). The pitch of chromatin DNA is reflected in its nucleotide sequence.. Proceedings of the National Academy of Sciences of the United States of America.

[18] Schieg P, Herzel H (2004). Periodicities of 10–11 bp as indicators of the supercoiled state of genomic DNA.. Journal of Molecular Biology.

[19] Hosid S, Trifonov EN, Bolshoy A (2004). Sequence periodicity of Escherichia coli is concentrated in intergenic regions.. BMC molecular biology.

[20] Holste D, Grosse I, Beirer S, Schieg P, Herzel H (2003). Repeats and correlations in human DNA sequences.. Physical review E, Statistical, nonlinear, and soft matter physics.

[21] Genome Assembly/Annotation Projects of The NCBI ftp site.. ftp://ftp.ncbi.nih.gov/genomes/.

[22] Sharp PM, Li W-H (1987). The codon adaptation index-a measure of directional synonymous codon usage bias, and its potential applications.. Nucl Acids Res.

[23] Tatusov RL, Koonin EV, Lipman DJ (1997). A Genomic Perspective on Protein Families.. Science.

[24] Schneider KL, Pollard KS, Baertsch R, Pohl A, Lowe TM (2006). The UCSC Archaeal Genome Browser.. Nucleic Acids Res.

[25] Shpigelman ES, Trifonov EN, Bolshoy A (1993). CURVATURE: software for the analysis of curved DNA.. Bioinformatics.

[26] NCBI COGs.. http://www.ncbi.nlm.nih.gov/COG/.

[27] Lobry JR (1996). Asymmetric substitution patterns in the two DNA strands of bacteria.. Mol Biol Evol.

[28] Freeman JM, Plasterer TN, Smith TF, Mohr SC (1998). Patterns of genome organization in bacteria.. Science.

[29] Grigoriev A (1998). Analyzing genomes with cumulative skew diagrams.. Nucl Acids Res.

[30] McLean MJ, Wolfe KH, Devine KM (1998). Base composition skews, replication orientation, and gene orientation in 12 prokaryote genomes.. Journal of Molecular Evolution.

[31] Tillier ERM, Collins RA (2000). The Contributions of Replication Orientation, Gene Direction, and Signal Sequences to Base-Composition Asymmetries in Bacterial Genomes.. Journal of Molecular Evolution.

[32] Rocha EPC (2004). The replication-related organization of bacterial genomes.. Microbiology.

[33] Mackiewicz P, Zakrzewska-Czerwinska J, Zawilak A, Dudek MR, Cebrat S (2004). Where does bacterial replication start? Rules for predicting the oriC region.. Nucl Acids Res.

[34] Worning P, Jensen LJ, Hallin PF, Staerfeldt HH, Ussery DW (2006). Origin of replication in circular prokaryotic chromosomes.. Environmental Microbiology.

[35] Gao F, Zhang CT (2007). DoriC: a database of oriC regions in bacterial genomes.. Bioinformatics.

[36] Sernova NV, Gelfand MS (2008). Identification of replication origins in prokaryotic genomes.. Brief Bioinform.

[37] Chen C, Chen CW (2007). Quantitative analysis of mutation and selection pressures on base composition skews in bacterial chromosomes.. BMC Genomics.

[38] Touzain F, Petit MA, Schbath S, Karoui ME (2011). DNA motifs that sculpt the bacterial chromosome.. Nat Rev Microbiol.

[39] Trivedi S, Rao SR, Gehlot HS (2005). Nucleic acid stability in thermophilic prokaryotes: a review.. Journal of Cell and Molecular Biology.

[40] Fugal DL (2009).

[41] Holland RC, Down TA, Pocock M, Prlic A, Huen D (2008). BioJava: an open-source framework for bioinformatics.. Bioinformatics.

[42] Friedel M, Nikolajewa S, Suhnel J, Wilhelm T (2009). DiProDB: a database for dinucleotide properties.. Nucleic Acids Res.

[43] Hsiao W, Wan I, Jones SJ, Brinkman FS (2003). IslandPath: aiding detection of genomic islands in prokaryotes.. Bioinformatics.

[44] Waack S, Keller O, Asper R, Brodag T, Damm C (2006). Score-based prediction of genomic islands in prokaryotic genomes using hidden Markov models.. BMC Bioinformatics.

[45] SVG, Scalale Vector Graphics.. http://www.w3.org/TR/SVG/.

[46] DOM, Document Object Model.. http://www.w3.org/DOM/.

[47] R Development Core Team (2011). R: A language and environment for statistical computing. R Foundation for Statistical Computing, Vienna, Austria. ISBN 3-900051-07-0.. http://www.R-project.org.

[48] Urbanek S (2010). Rserve: Binary R server.. http://CRAN.R-project.org/package=Rserve.

[49] Whitcher B, Guttorp P, Percival D (2000). Wavelet analysis of covariance with application to atmospheric time series.. Journal of Geophysical Research.

[50] Whitcher B (2009). waveslim: Basic wavelet routines for one-, two- and three-dimensional signal processing.. http://CRAN.R-project.org/package=waveslim.

[51] Chatfield C (1980). The analysis of time series: an introduction.

[52] Levine JD, Funes P, Dowse HB, Hall JC (2002). Signal analysis of behavioral and molecular cycles.. BMC Neurosci.

