# Supplementary figures and images for: CAGO: A Software Tool for Dynamic Visual Comparison and Correlation Measurement of Genome Organization
Source: PLoS One. 2011 Nov 17;6(11):e27080. doi: 10.1371/journal.pone.0027080 (PMC3219657; doi:10.1371/journal.pone.0027080)

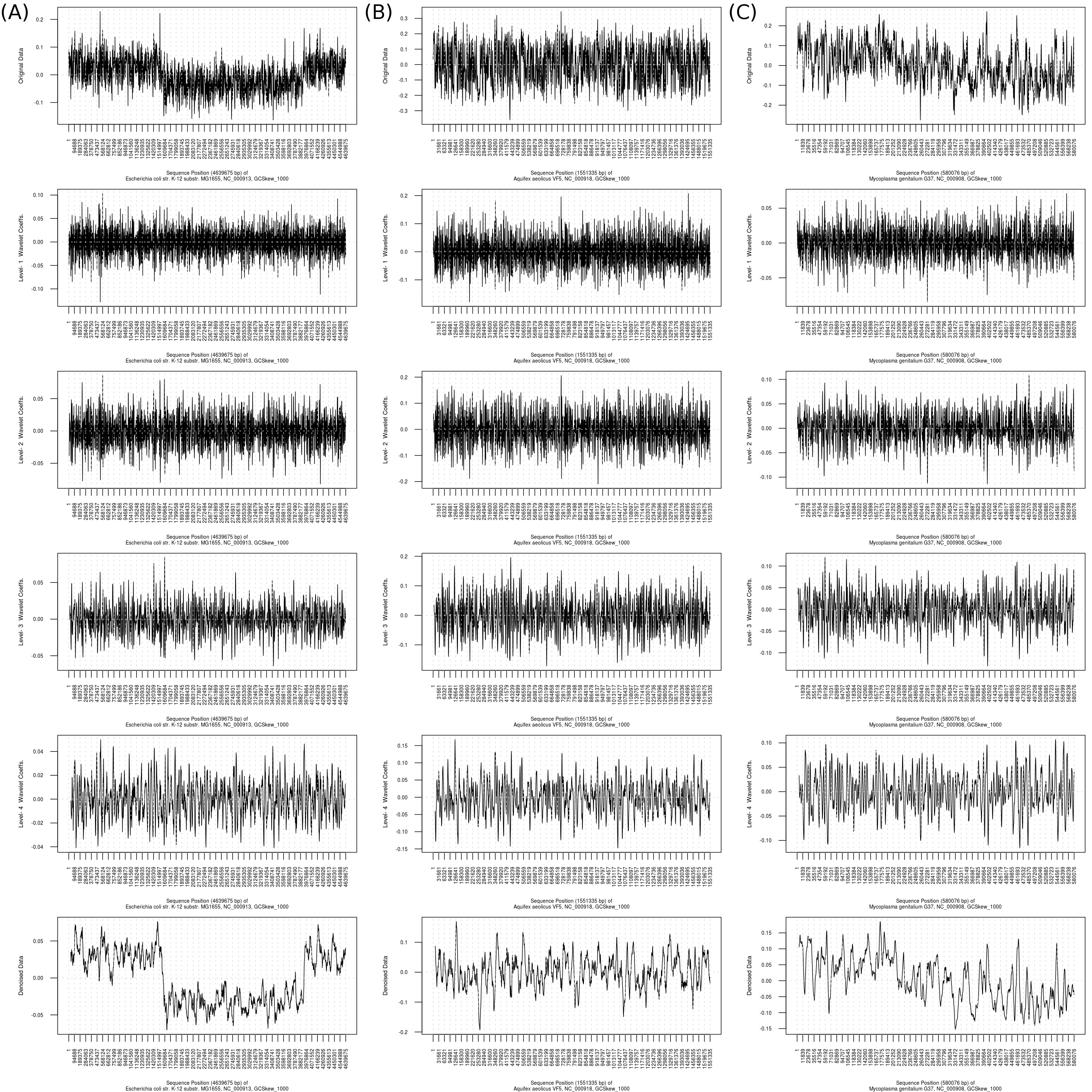

Supplement: Figure S1 — Results of discrete wavelet transformation for the GC skews of the chromosomes of E. coli , A. aeolicus and M. genitalium . Figure S1 shows the plots of original GC skews, the plots of level-1 to level-4 wavelet coefficients, and the plots of the denoised versions of GC skews for the chromosomes of (A) E. coli, (B) A. aeolicus and (C) M. genitalium, respectively. The X-axis represents the sequence positions of each organism. The Y-axis represents the values of a GC skew or the values of wavelet coefficients. (TIFF) [file pone.0027080.s001.tiff]

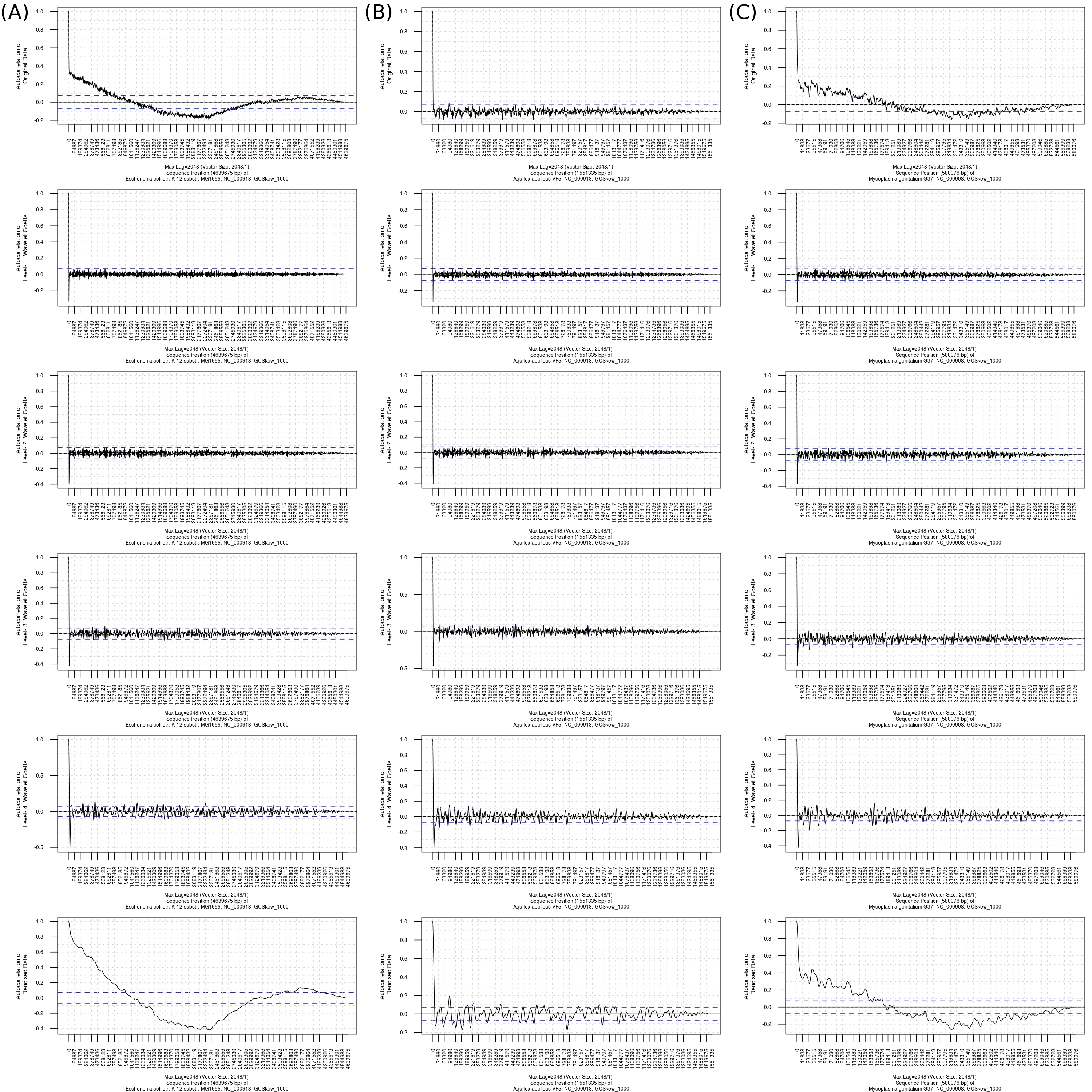

Supplement: Figure S2 — Results of autocorrelation analysis for the GC skews of the chromosomes of E. coli , M. genitalium and A. aeolicus . Figure S2 shows the results of autocorrelation analysis on the original GC skews, the wavelet coefficients and the denoised versions for the GC skews of the chromosomes of (A) E. coli, (B) A. aeolicus and (C) M. genitalium, respectively. The X-axis represents the shifting lags (sequence position) of each organism. The Y-axis represents the degrees of autocorrelation coefficients at different lags. The two horizontal blue lines are the 99.9% of confidence interval (0.073). (TIFF) [file pone.0027080.s002.tiff]
