# Supplementary material for: CAGO: A Software Tool for Dynamic Visual Comparison and Correlation Measurement of Genome Organization
Source: PLoS One. 2011 Nov 17;6(11):e27080. doi: 10.1371/journal.pone.0027080 (PMC3219657; doi:10.1371/journal.pone.0027080)
Supplement: Table S1 — Feature comparison of genome atlas plotters. (DOC) [file pone.0027080.s003.doc]

## Table S1. Feature Comparison of Genome Atlas Viewers

| Tools | Type of Map | | Comparative Genomics | | | Interactivity | | | Data Source | | |
| --- | --- | --- | --- | --- | --- | --- | --- | --- | --- | --- | --- |
|  | Circular | Linear | Genometric  Comparison | Sequence  Comparison^1^ | Multiple Chromosome on Single Map | GUI for  Constructing New Map | Zooming | Dynamic Track  Manipulation | Pre-Computed Data | Standard Sequence  File Formats | Customized  Data |
| CAGO | ✔ | ✔ | ✔ | ✔ | ✔ | Web | ✔^2^ |  | ✔ | GenBank, PTT | ✔ |
| GenomePlot | ✔ | ✔ |  | ✔ | ✔ | CLI (Perl/TK) |  |  |  |  | ✔ |
| GenoMap | ✔ |  |  | ✔ |  | Tcl/Tk-based |  |  |  |  | ✔ |
| Genome Atlas | ✔ |  |  |  |  | Web Service |  |  | ✔^3^ | GenBank, FASTA |  |
| ChromoViz |  | ✔ |  | ✔ | ✔ | Web, R package | ✔ |  | ✔^5^ | GFF | ✔ |
| Microbial Genome Viewer | ✔ | ✔^4^ |  |  | ✔ | Web |  |  | ✔ | EMBL, GenBank | ✔ |
| CGView | ✔ |  |  | ✔ |  | Web, CLI | ✔ |  | ✔ | PTT | ✔ |
| BacMap | ✔ |  |  |  |  | Web | ✔ |  | ✔^3^ |  |  |
| GenomeDiagram | ✔ | ✔ |  | ✔ | ✔ | Python Module |  |  |  | EMBL, GenBank |  |
| Circos | ✔ |  |  | ✔ | ✔ | CLI (Perl) |  |  |  |  | ✔ |
| 3D genome tuner | ✔ | ✔ |  | ✔ | ✔ | Java | ✔ |  |  | EMBL, GenBank, FASTA, BLAST |  |
| Genome Projector | ✔ | ✔ |  |  |  |  | ✔ |  | ✔^3^ |  |  |
| DNAPlotter | ✔ | ✔ |  | ✔ |  | Java | ✔ |  | ✔ | EMBL, GenBank, GFF | ✔ |
| GeneWiz | ✔ | ✔ | ✔ | ✔ |  | Web | ✔ |  | ✔ | GenBank, FASTA | ✔ |
| GView | ✔ | ✔ |  | ✔ |  | Web (Java Web Start), CLI, API | ✔ |  |  | EMBL, GenBank, GFF | ✔ |
| Easyfig |  | ✔ |  | ✔ | ✔ | Python/CLI | ✔ |  |  | GenBank, EMBL, BLAST |  |
| Gobe |  | ✔ |  | ✔ | ✔ | Web (Flash), Web Service, | ✔ | ✔ | ✔ | BED, GFF, BLAST | ✔ |
| Jena Prokaryotic Genome Viewer | ✔ | ✔^4^ |  | ✔ | ✔ | Web |  |  | ✔ |  |  |

^1^: Present sequence comparison results as a track of chromosome map

^2^: Requires Adobe SVG viewer

^3^: Only pre-computed static images are available

^4^: Use linear chromosome viewer as genome annotation browser

^5^: Only Human and Mouse genomes are available

API: Application Programming Interface

CLI: command-line interface

PTT: NCBI protein table file

BED: http://genome.ucsc.edu/FAQ/FAQformat#format1
