# Supplementary material for: CAGO: A Software Tool for Dynamic Visual Comparison and Correlation Measurement of Genome Organization
Source: PLoS One. 2011 Nov 17;6(11):e27080. doi: 10.1371/journal.pone.0027080 (PMC3219657; doi:10.1371/journal.pone.0027080)
Supplement: Table S2 — Available genomic features and genomic properties in CAGO. (DOC) [file pone.0027080.s004.doc]

## Table S2. Available Genomic Features and Genometric Properties in CAGO

| Name of Genomic Features and Genometric Properties | (Genomic Features/ Genometric Properties) | Source |
| --- | --- | --- |
| CDS_Forward | (/-) | Annotations |
| CDS_Reverse | (/-) |
| CDS_Density | (/-) |
| tRNA | (/-) |
| rRNA | (/-) |
| COG-All COG Functional Categroies | (/-) | Annotations |
| J-Translation | (/-) |
| A-RNA processing and modification | (/-) |
| K-Transcription | (/-) |
| L-Replication, recombination and repair | (/-) |
| B-Chromatin structure and dynamics | (/-) |
| D-Cell cycle control, mitosis and meiosis | (/-) |
| Y-Nuclear structure | (/-) |
| V-Defense mechanisms | (/-) |
| T-Signal transduction mechanisms | (/-) |
| M-Cell wall/membrane biogenesis | (/-) |
| N-Cell motility | (/-) |
| Z-Cytoskeleton | (/-) |
| W-Extracellular structures | (/-) |
| U-Intracellular trafficking and secretion | (/-) |
| O-Posttranslational modification, protein turnover, chaperones | (/-) |
| C-Energy production and conversion | (/-) |
| G-Carbohydrate transport and metabolism | (/-) |
| E-Amino acid transport and metabolism | (/-) |
| F-Nucleotide transport and metabolism | (/-) |
| H-Coenzyme transport and metabolism | (/-) |
| I-Lipid transport and metabolism | (/-) |
| P-Inorganic ion transport and metabolism | (/-) |
| Q-Secondary metabolites biosynthesis, transport and catabolism | (/-) |
| R-General function prediction only | (/-) |
| S-Function unknown | (/-) |
| Genomic Island: IslandPath (Bacteria only) | (/-) | Sequence |
| Genomic Island: SIGIHMM (Bacteria only) | (/-) |
| CAI: Codon Adaption Index | (-/) |
| Percent A (window size: 100bp/1kb) | (-/) |
| Percent C (window size: 100bp/1kb) | (-/) |
| Percent G (window size: 100bp/1kb) | (-/) |
| Percent T (window size: 100bp/1kb) | (-/) |
| Percent AC (window size: 100bp/1kb) | (-/) |
| Percent AG (window size: 100bp/1kb) | (-/) |
| Percent AT (window size: 100bp/1kb) | (-/) |
| Percent GC (window size: 100bp/1kb) | (-/) |
| Percent GT (window size: 100bp/1kb) | (-/) |
| Percent CT (window size: 100bp/1kb) | (-/) |
| AG Skew (A-G)/(A+G) (window size: 100bp/1kb) | (-/) |
| AC Skew (A-C)/(A+C) (window size: 100bp/1kb) | (-/) |
| AT Skew (A-T)/(A+T) (window size: 100bp/1kb) | (-/) |
| GC Skew (G-C)/(G+C) (window size: 100bp/1kb) | (-/) |
| GT Skew (G-T)/(G+T) (window size: 100bp/1kb) | (-/) |
| CT Skew (C-T)/(C+T) (window size: 100bp/1kb) | (-/) |
| Weak Skew (AT-GC)/(A+C+G+T) (window size: 100bp/1kb) | (-/) |
| Strong Skew (GC-AT)/(A+C+G+T) (window size: 100bp/1kb) | (-/) |
| Purine Skew (AG-CT)/(A+C+G+T) (window size: 100bp/1kb) | (-/) |
| Pyrimidin Skew (CT-AG)/(A+C+G+T) (window size: 100bp/1kb) | (-/) |
| Amino Skew (AC-GT)/(A+C+G+T) (window size: 100bp/1kb) | (-/) |
| Keto Skew (GT-AC)/(A+C+G+T) (window size: 100bp/1kb) | (-/) |
| Cumulative AC Skew (window size: 100bp/1kb) | (-/) |
| Cumulative AG Skew (window size: 100bp/1kb) | (-/) |
| Cumulative AT Skew (window size: 100bp/1kb) | (-/) |
| Cumulative GC Skew (window size: 100bp/1kb) | (-/) |
| Cumulative GT Skew (window size: 100bp/1kb) | (-/) |
| Cumulative CT Skew (window size: 100bp/1kb) | (-/) |
| Nucleosome Position Preference | (-/) |  |
| DNAseI Sensitivity | (-/) |  |
| Protein Deformability | (-/) |  |
| Curvature | (-/) |  |
| Bend (Karas 1996) | (-/) | Diprodb |
| Clash Strength (Gorin 1995) | (-/) |
| Direction (Shpigelman 1993) | (-/) |
| Inclination (Karas1996) | (-/) |
| Persistance Length (Hogan 1987) | (-/) |
| Probability contacting nucleosome core (Hogan 1987) | (-/) |
| Tip (Karas 1996) | (-/) |
| Wedge (Shpigelman 1993) | (-/) |
| Enthalpy (Sugimoto 1996) | (-/) |
| Enthalpy RNA (Freier 1986) | (-/) |
| Enthalpy RNA (Xia 1998) | (-/) |
| Entropy (Sugimoto 1996) | (-/) |
| Entropy RNA (Freier 1986) | (-/) |
| Entropy RNA (Xia 1998) | (-/) |
| Free energy (Gotoh 1981) | (-/) |
| Free energy (Vologodskii 1984) | (-/) |
| Free energy (Breslauer 1986) | (-/) |
| Free energy (Delcourt 1991) | (-/) |
| Free energy (Owczarzy 1992) | (-/) |
| Free energy (Sugimoto 1996) | (-/) |
| Free energy (SantaLucia 1996) | (-/) |
| Free energy (Allawi 1997) | (-/) |
| Free energy RNA (Freier 1986) | (-/) |
| Free energy RNA (Xia 1998) | (-/) |
| Hydrophilicity RNA (Barzilay 1973) | (-/) |
| Hydrophilicity RNA (Weber 1978) | (-/) |
| Major Groove Depth (Karas 1996) | (-/) |
| Major Groove Distance (Gorin 1995) | (-/) |
| Major Groove Size (Gorin 1995) | (-/) |
| Major Groove Width (Karas 1996) | (-/) |
| Minor Groove Depth (Karas 1996) | (-/) |
| Minor Groove Distance (Gorin 1995) | (-/) |
| Minor Groove Size (Gorin 1995) | (-/) |
| Minor Groove Width (Karas 1996) | (-/) |
| Mobility to bend towards major groove (Gartenberg 1988) | (-/) |
| Mobility to bend towards minor groove (Gartenberg 1988) | (-/) |
| Melting Temp. (Gotoh 1981) | (-/) |
| Melting Temp. (Anselmi 2002) | (-/) |
| Rise (Karas 1996) | (-/) |
| Rise (Suzuki 1996) | (-/) |
| Rise (Perez 2004) | (-/) |
| Rise DNA protein complex (Suzuki 1996) | (-/) |
| Rise DNA protein complex (Olson 1998) | (-/) |
| Rise RNA (Perez 2004) | (-/) |
| Rise stiffness (Goni 2007) | (-/) |
| Rise rise (Lankas 2003) | (-/) |
| Roll (Gorin 1995) | (-/) |
| Roll (Suzuki 1996) | (-/) |
| Roll (Anselmi 2002) | (-/) |
| Roll (Perez 2004) | (-/) |
| Roll DNA protein complex (Suzuki 1996) | (-/) |
| Roll DNA protein complex (Olson 1998) | (-/) |
| Roll RNA (Perez 2004) | (-/) |
| Roll stiffness (Goni 2007) | (-/) |
| Roll rise (Lankas 2003) | (-/) |
| Roll roll (Lankas 2003) | (-/) |
| Roll shift (Lankas 2003) | (-/) |
| Roll slide (Lankas 2003) | (-/) |
| Shift (Suzuki 1996) | (-/) |
| Shift (Perez 2004) | (-/) |
| Shift DNA protein complex (Suzuki 1996) | (-/) |
| Shift DNA protein complex (Olson 1998) | (-/) |
| Shift RNA (Perez 2004) | (-/) |
| Shift stiffness (Goni 2007) | (-/) |
| Shift rise (Lankas 2003) | (-/) |
| Shift shift (Lankas 2003) | (-/) |
| Shift slide (Lankas 2003) | (-/) |
| Slide (Gorin 1995) | (-/) |
| Slide (Suzuki 1996) | (-/) |
| Slide (Perez 2004) | (-/) |
| Slide DNA protein complex (Suzuki 1996) | (-/) |
| Slide DNA protein complex (Olson 1998) | (-/) |
| Slide RNA (Perez 2004) | (-/) |
| Slide stiffness (Goni 2007) | (-/) |
| Slide rise (Lankas 2003) | (-/) |
| Slide slide (Lankas 2003) | (-/) |
| Stacking energy (Sponer 1997) | (-/) |
| Stacking energy (Ussery 2002) | (-/) |
| Stacking energy (Anselmi 2002) | (-/) |
| Stacking energy (Perez 2004) | (-/) |
| Stacking energy (RNA Perez 2004) | (-/) |
| Tilt (Gorin 1995) | (-/) |
| Tilt (Suzuki 1996) | (-/) |
| Tilt (Anselmi 2002) | (-/) |
| Tilt (Perez 2004) | (-/) |
| Tilt DNA protein complex (Suzuki 1996) | (-/) |
| Tilt DNA protein complex (Olson 1998) | (-/) |
| Tilt RNA (Perez 2004) | (-/) |
| Tilt stiffness (Goni 2007) | (-/) |
| Tilt rise (Lankas 2003) | (-/) |
| Tilt roll (Lankas 2003) | (-/) |
| Tilt shift (Lankas 2003) | (-/) |
| Tilt slide (Lankas 2003) | (-/) |
| Tilt tilt (Lankas 2003) | (-/) |
| Twist (Shpigelman 1993) | (-/) |
| Twist (Gorin 1995) | (-/) |
| Twist (Karas 1996) | (-/) |
| Twist (Suzuki 1996) | (-/) |
| Twist (Anselmi 2002) | (-/) |
| Twist (Perez 2004) | (-/) |
| Twist DNA protein complex (Suzuki 1996) | (-/) |
| Twist DNA protein complex (Olson 1998) | (-/) |
| Twist RNA (Perez 2004) | (-/) |
| Twist stiffness (Goni 2007) | (-/) |
| Twist rise (Lankas 2003) | (-/) |
| Twist roll (Lankas 2003) | (-/) |
| Twist shift (Lankas 2003) | (-/) |
| Twist slide (Lankas 2003) | (-/) |
| Twist tilt (Lankas 2003) | (-/) |
| Twist twist (Lankas 2003) | (-/) |
| Propeller Twist (Gorin 1995) | (-/) |
| M3D Microarray data for E. coli | (-/) |  |
| Conserved in Class Acidobacteria | (-/) | All against All Blastp (identity >= 25%, Coverage >= 40%) |
| Conserved in Class Actinobacteria | (-/) |
| Conserved in Class Alphaproteobacteria | (-/) |
| Conserved in Class Betaproteobacteria | (-/) |
| Conserved in Class Deltaproteobacteria | (-/) |
| Conserved in Class Epsilonproteobacteria | (-/) |
| Conserved in Class Gammaproteobacteria | (-/) |
| Conserved in Class Aquificae | (-/) |
| Conserved in Class Archaeoglobi | (-/) |
| Conserved in Class Bacilli | (-/) |
| Conserved in Class Bacteroidia | (-/) |
| Conserved in Class Chlamydiae | (-/) |
| Conserved in Class Chlorobia | (-/) |
| Conserved in Class Chloroflexi | (-/) |
| Conserved in Class Clostridia | (-/) |
| Conserved in Class Deferribacteres | (-/) |
| Conserved in Class Dehalococcoidetes | (-/) |
| Conserved in Class Deinococci | (-/) |
| Conserved in Class Dictyoglomia | (-/) |
| Conserved in Class Elusimicrobia | (-/) |
| Conserved in Class Fibrobacteres | (-/) |
| Conserved in Class Flavobacteria | (-/) |
| Conserved in Class Fusobacteria | (-/) |
| Conserved in Class Gemmatimonadetes | (-/) |
| Conserved in Class Gloeobacteria | (-/) |
| Conserved in Class Halobacteria | (-/) |
| Conserved in Class Methanobacteria | (-/) |
| Conserved in Class Methanococci | (-/) |
| Conserved in Class Methanomicrobia | (-/) |
| Conserved in Class Methanopyri | (-/) |
| Conserved in Class Mollicutes | (-/) |
| Conserved in Class Nitrospira | (-/) |
| Conserved in Class Opitutae | (-/) |
| Conserved in Class Planctomycetacia | (-/) |
| Conserved in Class Solibacteres | (-/) |
| Conserved in Class Sphingobacteria | (-/) |
| Conserved in Class Spirochaetes | (-/) |
| Conserved in Class Synergistia | (-/) |
| Conserved in Class Thermococci | (-/) |
| Conserved in Class Thermomicrobia | (-/) |
| Conserved in Class Thermoplasmata | (-/) |
| Conserved in Class Thermoprotei | (-/) |
| Conserved in Class Thermotogae | (-/) |
| Conserved in Class Verrucomicrobiae | (-/) |

References

1. Hsiao W, Wan I, Jones SJ, Brinkman FS (2003) IslandPath: aiding detection of genomic islands in prokaryotes. Bioinformatics 19: 418-420.

2. Waack S, Keller O, Asper R, Brodag T, Damm C, et al. (2006) Score-based prediction of genomic islands in prokaryotic genomes using hidden Markov models. BMC Bioinformatics 7: 142.

3. Pedersen AG, Baldi P, Chauvin Y, Brunak S (1998) DNA structure in human RNA polymerase II promoters. Journal of Molecular Biology 281: 663-673.

4. Brukner I, Sanchez R, Suck D, Pongor S (1995) Sequence-dependent bending propensity of DNA as revealed by DNase I: parameters for trinucleotides. The EMBO journal 14: 1812-1818.

5. Olson WK, Gorin AA, Lu XJ, Hock LM, Zhurkin VB (1998) DNA sequence-dependent deformability deduced from protein-DNA crystal complexes. Proceedings of the National Academy of Sciences of the United States of America 95: 11163-11168.

6. Shpigelman ES, Trifonov EN, Bolshoy A (1993) CURVATURE: software for the analysis of curved DNA. Bioinformatics 9: 435-440.

7. Friedel M, Nikolajewa S, Suhnel J, Wilhelm T (2009) DiProDB: a database for dinucleotide properties. Nucleic Acids Res 37: D37-40.

8. Faith JJ, Driscoll ME, Fusaro VA, Cosgrove EJ, Hayete B, et al. (2008) Many Microbe Microarrays Database: uniformly normalized Affymetrix compendia with structured experimental metadata. Nucleic Acids Research 36: D866-870.
